# From the Mind to the Spine: The Intersecting World of Alzheimer’s and Osteoporosis

**DOI:** 10.1007/s11914-023-00848-w

**Published:** 2024-02-09

**Authors:** Tyler J. Margetts, Hannah S. Wang, Sonali J. Karnik, Lilian I. Plotkin, Alexandru Movila, Adrian L. Oblak, Jill C. Fehrenbacher, Melissa A. Kacena

**Affiliations:** 1 Department of Orthopaedic Surgery, Indiana University School of Medicine, Indianapolis, IN 46202 USA; 2 Department of Anatomy, Cell Biology & Physiology, Indiana University School of Medicine, Indianapolis, IN 46202 USA; 3 Indiana Center for Musculoskeletal Health, Indiana University School of Medicine, Indianapolis, IN USA; 4 Richard L. Roudebush VA Medical Center, Indianapolis, IN 46202 USA; 5 Department of Biomedical Sciences and Comprehensive Care, Indiana University School of Dentistry, Indianapolis, IN 46202 USA; 6 Department of Radiology & Imaging Sciences, Stark Neurosciences Research Institute, Indiana University School of Medicine, Indianapolis, IN 46202 USA; 7 Stark Neurosciences Research Institute, Indiana University School of Medicine, Indianapolis, IN 46202 USA; 8 Department of Pharmacology and Toxicology, Indiana University School of Medicine, Indianapolis, IN 46202 USA

**Keywords:** Osteoporosis, Alzheimer’s, Bone, Fracture, Neuroinflammation, Estrogen, AI, Artificial intelligence, ChatGPT

## Abstract

**Purpose of Review:**

This comprehensive review delves into the intricate interplay between Alzheimer’s disease (AD) and osteoporosis, two prevalent conditions with significant implications for individuals’ quality of life. The purpose is to explore their bidirectional association, underpinned by common pathological processes such as aging, genetic factors, inflammation, and estrogen deficiency.

**Recent Findings:**

Recent advances have shown promise in treating both Alzheimer’s disease (AD) and osteoporosis by targeting disease-specific proteins and bone metabolism regulators. Monoclonal antibodies against beta-amyloid and tau for AD, as well as RANKL and sclerostin for osteoporosis, have displayed therapeutic potential. Additionally, ongoing research has identified neuroinflammatory genes shared between AD and osteoporosis, offering insight into the interconnected inflammatory mechanisms. This knowledge opens avenues for innovative dual-purpose therapies that could address both conditions, potentially revolutionizing treatment approaches for AD and osteoporosis simultaneously.

**Summary:**

This review underscores the potential for groundbreaking advancements in early diagnosis and treatment by unraveling the intricate connection between AD and bone health. It advocates for a holistic, patient-centered approach to medical care that considers both cognitive and bone health, ultimately aiming to enhance the overall well-being of individuals affected by these conditions. This review article is part of a series of multiple manuscripts designed to determine the utility of using artificial intelligence for writing scientific reviews.

## Introduction

This is one of many articles evaluating the assistance of using AI to write scientific review articles on musculoskeletal topics [1]. The first draft of this review was written by humans and ChatGPT4.0 whereby humans selected literature references, but ChatGPT 4.0 completed the writing. Importantly, the article was edited and carefully checked for accuracy resulting in a final manuscript which was significantly different from the original draft. Refer to this edition’s Comment paper for more information [2]. In the realm of geriatric health, the intricate interplay between Alzheimer’s disease (AD), osteoporosis, and fractures presents a compelling area of study. Aging has been shown to be a significant risk factor for each of these pathologies, and the steady increase in world life expectancy creates a need for further understanding of them [3–6]. While individually significant, these health conditions collectively form a complex tapestry of interrelated pathophysiological mechanisms [7–9].

AD, a neurodegenerative disorder, has been linked to an increased risk of fractures. Individuals with Alzheimer’s disease have an increased risk (some report up to three times) of sustaining a hip fracture, underscoring the profound impact of cognitive disorders on physical health [7, 10, 11]. Conversely, a history of fractures has been identified as an independent risk factor for the development of dementia, with AD being the most common form of cognitive decline [7, 8]. This suggests a bidirectional relationship that warrants further exploration. Building on these studies, the relationship between AD and bone extends to osteoporosis, a condition characterized by low bone mass and structural deterioration of bone tissue [12]. Multiple mouse models of AD have been shown to express an osteoporotic phenotype [13•, 14–17, 18••]. Osteoporosis is a well-known risk factor for fractures, but intriguingly, recent research has also linked bone loss to subsequent cognitive decline, further emphasizing the interconnectedness of these conditions [9].

In this review, we aim to unravel the complex relationship between AD, osteoporosis, and fracture. By delving into the shared mechanisms and exploring potential therapeutic targets, we hope to pave the way for novel interventions that address these conditions, ultimately improving outcomes for our aging population.

## Background on Alzheimer’s Disease

AD is a pervasive neurodegenerative disorder that poses a significant public health challenge. As of 2023, it is estimated that between 10 to 12 million older Americans are living with AD and some form of cognitive deficits. Specific symptoms and speed of progression are variable among patients, but typically the disease presents as an impairment in memory, language, and thinking [19]. The prevalence of AD and related dementias increases with age, with the disease affecting approximately 3% of individuals aged 65–74, 17% of individuals aged 75–84, and 32% of individuals aged 85 and older [19]. The economic burden of AD is also substantial, with the total estimated costs of health care, long-term care, and hospice for people with AD and other dementias at 345 billion dollars in the USA in 2023. This number is projected to increase to more than $1.1 trillion by 2050 [19].

Among those patients with AD, records show that only a very small percent of cases suffer from familial AD (fAD), with the majority caused by missense mutations in the presenilin 1 (*PSEN1*) gene, *PSEN2* gene, and in the Amyloid Precursor Protein (*APP*) gene. This form of the disease can present earlier in life and is inherited in an autosomal dominant pattern. Most of AD patients, comprising over 95% of cases, are designated as Late-Onset Alzheimer’s Disease (LOAD) [20, 21]. The non-familial form of AD will be referred to as “AD” for the remainder of this review.

The risk factors for AD are multifaceted, encompassing lifestyle factors, age, sex, family history, and genetics. In particular, the apolipoprotein E4 variant of the *APOE* gene is one of the most significant genetic risk factors for AD, as over 60% of people with the disease carry at least one copy of the allele [22, 23]. Lifestyle factors such as diet, exercise, and cognitive training also play a role in maintaining cognitive function and may potentially help in preventing AD [24].

The main neuropathological hallmarks of AD are the accumulation of extracellular amyloid-beta (Aβ) plaques and intracellular neurofibrillary tangles (NFTs) [20, 22, 25–30]. Aβ plaques are primarily composed of Aβ peptides, which are generated from the sequential cleavage of the APP by β- and γ-secretases [22, 26, 27, 29]. These peptides are prone to aggregation, forming the Aβ plaques which can impair cognitive function and cause neurotoxicity [20, 22, 24, 29].

On the other hand, NFTs are composed of hyperphosphorylated tau protein. Under normal physiological conditions, tau protein promotes microtubule assembly and stability. However, in AD, tau undergoes abnormal hyperphosphorylation, leading to the destabilization of microtubules and aggregation of NFTs [30]. The formation of these tangles is thought to be a result of an imbalance between tau phosphorylation and dephosphorylation, with several kinases and phosphatases implicated in this process [24, 25, 30].

It has been proposed that tau pathology and Aβ plaques stimulate an inflammatory response by the nervous system through the activation of microglia and astrocytes in response to neuronal injury, resulting in production of cytokines, chemokines, and reactive oxygen species (ROS) [31]. This neuroinflammation in turn causes increased formation of tau and Aβ pathology, creating a positive feedback loop that leads to AD symptoms through increased neuronal apoptosis [30].

The blood brain barrier (BBB) represents a combination of dynamic physical and chemical boundaries that regulate communication between the central nervous system and the rest of the body [32]. It is made of capillary endothelium and tight junctions and has been described as the microvasculature of the brain [33]. The most prevalent genetic risk factor for AD, the APOE4 variant, has been linked to BBB breakdown through APOE4 expression in astrocytes and pericytes, cells in the capillaries that support endothelial cells and help maintain the BBB [34, 35]. This BBB breakdown in the hippocampus (HC) and parahippocampal gyrus (PHG), measured through dynamic contrast-enhanced magnetic resonance imaging (DCE-MRI), was associated with cognitive decline in APOE4 carriers, but not APOE4 homozygotes.

In a healthy brain, angiogenesis, the growth of new blood vessels from pre-existing ones, helps to provide oxygen and nutrients that neurons and other brain cells require to survive. Angiogenesis, in concert with a tightly regulated BBB, functions to maintain environmental homeostasis in the brain and clear away pathological debris [36]. Impairment of angiogenesis has been shown in the setting of AD, and Aβ has shown anti-angiogenic properties [28, 37]. It has been postulated that vascular dysfunction in the form of impaired Aβ clearance and BBB weakening may be involved in the underlying pathophysiology of AD [38].

Despite the significant progress made in understanding the pathological processes underlying AD, there remain important gaps in our knowledge. Research will continue to uncover the exact role of neuroinflammation, ROS, and other pathologies in the development of the disease. Further research into the molecular biology of Aβ and tau, as well as these other processes, will be crucial for the development of effective therapeutic strategies for AD [22, 25, 30].

## Background on Osteoporosis

Osteoporosis is a significant health issue, affecting over 13 million people in the USA alone [39]. The International Osteoporosis Foundation has highlighted the severity of this issue, noting that one in three women and one in five men over the age of 50 will sustain a fracture due to osteoporosis in their lifetime [12]. While much less than the costs associated with AD, osteoporosis still provides a substantial financial burden, with recent estimates predicting annual healthcare costs of 25 billion dollars by 2025 in the USA [40].

This disease is primarily classified into three types: postmenopausal osteoporosis (Type I), senile osteoporosis (Type II), and secondary osteoporosis which results from various diseases, medications, and lifestyle changes [12]. The underlying mechanism is rooted in an imbalance in the bone remodeling process. In this process, bone resorption surpasses bone formation, leading to a net loss of bone [12, 39]. This imbalance is influenced by a multitude of risk factors, including age, sex, low body mass index, previous fragility fracture, secondary causes of osteoporosis, parental history of hip fracture, current smoking, alcohol intake of three or more units daily, and rheumatoid arthritis, among others [12].

The impact of osteoporosis becomes evident when a fracture occurs. Osteoporotic bones are porous and have low bone mineral density (BMD), meaning that lower amounts of mechanical load can cause a fracture [12, 41]. These fractures often lead to chronic pain, disability, and in some cases, loss of life. Specifically, fractures of the hip have been linked to a significant increase in mortality rates within the first year following the fracture, with men experiencing a higher mortality rate than women [12]. Importantly, BMD, which contributes to approximately 70% of bone strength is measured using dual X-ray absorptiometry (DXA). A diagnosis of osteoporosis is given if a person’s BMD is 2.5 standard deviations or more below the average value for young healthy women (a *T*-score of < −2.5 SD) [12].

Osteoporosis is a major issue in the elderly population with substantial health and economic impacts. Early detection and intervention are crucial in disease management to prevent progression and fracture occurrence. This highlights the need for increased awareness among healthcare providers and the public and the urgency for further research into the disease.

## Increased Risk of Fractures Following Alzheimer’s Disease Diagnosis

Fractures, a common complication of osteoporosis, have been shown to be associated with AD. Individuals with AD are more than twice as likely to experience incident fractures than those without AD, an increase that was observed in the first year following AD diagnosis [42, 43]. A study done in the UK expanded on this, reporting a threefold increase in the incidence of hip fractures at any point in time among AD patients compared with patients without AD [11]. This increased risk was not only confined to the immediate period following an AD diagnosis but persisted throughout the disease course. Additionally, a higher post-fracture mortality rate was observed among AD patients, with 27.2% of AD patients and 13.6% of non-AD patients failing to survive more than 6 months post-fracture [11].

Gait dysfunction may have a significant role in this increased fracture risk. MRI studies have shown that generalized brain atrophy and white matter hyperintensities are associated with a decline in gait scores, which include a number of measured parameters such as rhythm, pace, and variability, indicating the nonspecific role of cognition in gait function [44]. Attention and executive function play crucial roles in gait control and the regulation of speed and variability, potentially underlying the specific role of cognitive function in gait. Impairment of cognition in AD could predispose patients to abnormal gait, leading to increased falls and fractures. It has been hypothesized that the nucleus basalis of Meynert, a major supplier of cholinergic signaling to the cerebral cortex, may be crucial to uncovering the relationship between AD and gait dysfunction [44].

Bone health is another critical factor contributing to the increased fracture risk in AD patients. Fracture in AD has been associated with low BMD, low concentrations of 25-hydroxyvitamin D, and low serum ionized calcium, factors that make these patients more susceptible to fracture in the first place [45, 46].

Despite the clear association between AD and increased fracture risk, the treatment of osteoporosis, a major contributor to fracture risk, is often inadequate. Only a quarter of osteoporotic patients receive calcium and vitamin D, and just 12.0% receive other osteoporosis medications prior to sustaining a fracture [47]. This under-treatment is not improved in high-risk populations, such as those with dementia and those with a previous fragility fracture, suggesting missed opportunities for delaying or preventing major osteoporotic fractures [47].

The increased risk of fractures following an AD diagnosis is a significant concern that requires a multifaceted approach, encompassing improved cognitive and gait function assessment, enhanced bone health management, and better osteoporosis treatment strategies.

## Impaired Bone Health Increases Risk of Developing AD

The relationship between BMD and AD further supports the relationship between AD and osteoporosis. An analysis of a multicenter study of osteoporotic fractures found that women in the lowest quartile of BMD had poorer age-adjusted baseline cognitive scores than women in the highest quartile [48]. Another study found that higher rates of bone loss were predictive of subsequent cognitive decline in older women, independent of baseline bone mass [9]. Similarly, a group in China showed that subjects with mild cognitive impairment (MCI) and low BMD were found to convert to AD at significantly higher rates than those with high BMD [49]. Further evidence of the association between BMD and AD comes from a community-based prospective cohort study, which found that elderly women in the lowest quartile of femoral neck BMD had more than twice the incidence of AD and all-cause dementia compared with those in higher quartiles [50]. Interestingly, this study did not find a relationship between BMD and the risk of AD in men, suggesting a potential role of gender in this association that will be discussed in a later subsection.

The relationship between fractures and the subsequent development of dementia or AD has been a topic of increasing interest in the medical community. A recent study reported an increased risk of dementia or AD in individuals with previous distal radius, hip, and spine fractures [51]. One potential mechanism underlying this association is the occurrence of postoperative delirium (POD), a condition that is often observed in patients following a major surgery [52]. In particular, in a cohort of patients with surgically repaired femoral neck fractures, the presence of POD was correlated with an increased risk of developing dementia within 3 years of the operation [53]. POD has also been associated with breakdown in the BBB, providing a link between a complication of fracture surgery and AD [54].

Other studies have shown that oxidative stress, which is known to play a crucial role in the pathogenesis of AD, increases during the first month after a fracture [55–58]. Inflammatory markers C-reactive protein (CRP) and interleukin-6 (IL-6), which are also often elevated following a fracture [59], have been associated with an increased risk of all-cause dementia. While these markers are not specific for AD, they may still have a role in predicting dementia onset in the community [60]. These findings suggest that the physiological response to fractures could potentially contribute to cognitive decline and development of AD; however, the relationship between fractures and the subsequent development of dementia/AD is complex and likely involves multiple interconnected mechanisms, including POD, oxidative stress, and inflammation. Further research is needed to fully elucidate these mechanisms and to develop effective strategies for preventing dementia/AD in individuals who have experienced fractures.

## Shared Pathways Between Alzheimer’s Disease and Osteoporosis

The intersection of AD and osteoporosis, two seemingly disparate conditions, is gaining increasing attention in the medical research community. Recent studies have shown that osteoporosis and bone fractures occur in AD patients at over twice the rate as similarly aged neurotypical adults [61]. This is not a result of disease-related immobility, as these conditions often precede the diagnosis of AD [61]. Another study conducted in Finland found that individuals with AD were twice as likely to have sustained a previous hip fracture. They were also more likely to experience a subsequent hip fracture in 4-year follow-up [43]. These studies indicate that the co-occurrence of these two diseases is not merely a coincidence of aging but rather evidence of causation or a manifestation of shared pathological mechanisms.

The Wnt/β-catenin signaling pathway has emerged as a significant shared mechanism between AD and osteoporosis. This pathway is known to facilitate bone formation and promote synaptogenesis in the brain [61]. In the context of bone health, the Wnt/β-catenin signaling pathway plays a pivotal role in maintaining normal bone homeostasis. Osteocyte-specific deletion of β-catenin, a key component of the Wnt pathway, leads to significant cortical and cancellous bone loss in both the appendicular and axial skeleton that can be attributed to increased osteoclastic bone resorption [61, 62]. Osteoclasts also utilize this pathway by secreting Wnt ligands and chemoattractants that support the bone remodeling process by stimulating the differentiation of osteoblasts [63].

The Wnt/β-catenin signaling pathway also plays a significant role in the pathogenesis of AD. This pathway is crucial for neuronal survival, neurogenesis, and the regulation of synaptic plasticity, all of which are processes that are disrupted in AD. Activation of the Wnt/β-catenin signaling pathway inhibits Aβ production and tau protein hyperphosphorylation in the brain, both of which are hallmarks of AD. However, this pathway is greatly suppressed in the AD brain due to multiple pathogenic mechanisms, including the downregulation of Wnt proteins and the upregulation of Wnt antagonist DKK1 [64]. This leads to unchecked production and accumulation of these pathological proteins, which in turn activate inflammatory pathways that further inhibit Wnt signaling, creating a dangerous feed-forward cycle of AD pathogenesis and Wnt deficit [61, 64].

The receptor for advanced glycation end products (RAGE) is expressed throughout the brain and has been found to interact with Aβ. Overexpression of this receptor in transgenic AD mice leads to increased neuroinflammation, higher levels of Aβ deposition, and neuronal damage [65]. RAGE is thought to be capable of acting as a receptor for Aβ and has been implicated in the transport of Aβ through the BBB [66]. A RAGE and Aβ interaction has been shown to induce gene regulation that ultimately disrupts tight junctions and increases permeability in endothelial cells, indicating that this facilitated transport of Aβ is associated with a breach of BBB integrity [67]. This receptor also works with Aβ to affect bone health, as RAGE is required for both RANKL and Aβ induced osteoclast differentiation [15, 68]. The Swedish mutation in the amyloid precursor protein gene (APPswe) is a familial gene mutation that upregulates production of Aβ [14, 15]. APP is found in many tissues outside of the brain such as the heart, muscle, adipose, and skin, and the increase in Aβ in APPswe mice has been shown to upregulate osteoclast differentiation through a RAGE-dependent mechanism [15, 69]. The presence of the APPswe mutation has also been shown to suppress osteoblast differentiation and bone formation, leading to a decrease in osteoblastogenesis and loss of trabecular bone mass. This decrease in osteoblast differentiation is accompanied by increased adipogenesis and elevated bone marrow fat, displaying a skeletal aging-like osteoporotic deficit [14]. This suggests that dysregulation of APP accelerates skeletal aging, which could be part of the underlying mechanism for the increased bone fracture rate in AD patients.

The intersection of AD and osteoporosis is a burgeoning area of research that holds promise for uncovering shared pathological mechanisms and potential therapeutic targets. The shared disruption of Wnt/β-catenin signaling, relevance of the RAGE receptor, and the role of APP in both conditions underscores the intricate interplay between the brain and the skeletal system.

## Angiogenesis

Angiogenesis is a critical physiological process with significant implications in various pathological conditions, including osteoporosis and AD [70–75]. In the context of AD, angiogenesis and its regulators play a complex role. Aβ peptides, which were previously discussed as a potential key player in the pathogenesis of AD, have been found to possess anti-angiogenic properties [37]. Another study demonstrated that mouse models of Aβ amyloidosis showed an impaired ability to form new capillaries from arterial explants [28]. Paradoxically, vascular endothelial growth factor (VEGF), a potent stimulator of angiogenesis, and two VEGF receptors FLT1 and FLT 4 are found in increased levels in prefrontal cortex tissue and cerebrospinal fluid (CSF) of AD patients [76]. This increase is thought to be a compensatory response to counter insufficient vascularity or reduced perfusion apparent in AD [75, 77]. A study in AD mouse models used fluorescence microscopy to show that increased VEGF-A signaling resulted in decreased cerebral blood flow, indicating that this phenomenon may be a result of impaired VEGF signaling in AD patients [78].

Shifting the focus to osteoporosis, angiogenesis is closely tied to bone remodeling and osteogenesis. The formation of new blood vessels is crucial during both primary bone development and fracture repair in adults [72–74]. Reduced or inadequate blood flow has been linked to impaired fracture healing and disorders of low bone mass such as osteoporosis [72]. Angiogenesis precedes osteogenesis, providing the nutrients, growth factors, and oxygen that support the formation and differentiation of osteoblasts and osteoclasts [72, 79]. Furthermore, the interaction between bone marrow endothelial cells and hematopoietic progenitor cells, mediated by molecules such as VCAM-1, VLA-4, and the chemokine SDF-1, plays a crucial role in angiogenesis [72, 80–82]. This interaction is particularly relevant in the context of fracture healing, where angiogenesis is a key component of the repair process [72, 73].

Both osteoporosis and AD share a common thread of impaired angiogenesis, but their relationship through this process has not been fully elucidated. Understanding these shared and distinct pathways could provide new insights into the pathogenesis of these diseases and reveal potential therapeutic targets.

## Role of Sex Hormones

AD and osteoporosis are two conditions that disproportionately affect women, particularly postmenopausal women. Two-thirds of all individuals with AD are females, and studies have shown a greater incidence of AD in women than men of the same age group with the greatest difference being shown in subjects older than 90 [83, 84]. Similarly, osteoporosis is a significant health issue among aging postmenopausal females, as rapid bone loss at a rate of 3–5% occurs in the 5–10 years after menopause [85]. Rates of osteoporosis in women approximately double every 5 years after menopause, reaching 50.3% at age 85 and older [86]. A common thread between these two conditions is the role of estrogens, a group of hormones that decline after menopause with estradiol as the most potent form. These hormones have been implicated in both cognitive function and bone health [83–90].

Estrogen receptors are highly expressed in the brain, and estrogens have been shown to be beneficial for brain tissue in animal models by promoting both the growth of cholinergic neurons and metabolism of APP [84]. Studies have shown that higher circulating estrogen concentrations, predominantly estradiol, are associated with a lower risk of cognitive decline in postmenopausal women [84, 88, 91]. This relationship involves the regulation of sex hormone-binding globulin (SHBG) concentrations. SHBG binds strongly and specifically to estradiol, reducing its ability to bind to receptors and initiate responses. Research shows that SHBG levels are significantly elevated in AD patients compared to controls, suggesting that bioavailable estradiol may be lower than in controls [92, 93]. Conversely, one study found that higher levels of estradiol were associated with a higher risk of dementia, further highlighting the complex relationship of estrogens and cognitive health [94].

Hormone replacement therapy (HRT), which typically involves the administration of estradiol, has been shown to have beneficial effects on cognitive function [83, 84, 87]. However, the effectiveness of HRT may be influenced by the timing of administration. Hormone therapy with estrogens early in menopause may be protective against AD later in life. Using this same therapy further from menopause onset and later into life does not offer the same protection and may even put patients at increased risk of AD. This is known as the “critical window hypothesis” [87]. Another proposed explanation for these findings states that if neurons are healthy when exposed to estrogen, their response promotes cognitive health and AD prevention. However, if neurons are already compromised through cognitive pathology, estrogen exposure may worsen cognitive function. This “healthy cell bias hypothesis” indicates that older women could potentially see cognitive benefits from HRT if they are healthy at the time of administration. Both hypotheses require additional large-scale studies prior to the implementation of this treatment into patient care [95, 96].

The role of hormones in the pathogenesis of AD is not limited to estrogens. Along with a decrease in estrogens, menopause also leads to an increase in follicle-stimulating hormone (FSH). A recent study used ovariectomy in mice to simulate menopause and show that FSH can accelerate deposition of Aβ and tau in cortical and hippocampal neurons. The use of an anti-FSH antibody resulted in reversal of neuropathology and cognitive decline in these mice, further emphasizing the role of FSH in the AD phenotype [97••].

In the context of osteoporosis, estrogens play a crucial role in bone remodeling, where the primary cell types involved in bone remodeling are osteoblasts, osteocytes, and osteoclasts [12, 85, 86, 98]. Estrogens help to regulate the activity and lifespan of these bone cells, and HRT in the form of estradiol supplementation is a treatment option for postmenopausal osteoporosis [86]. Although HRT provides an increase in bone density and reduction of fracture risk in postmenopausal women, it is typically considered after other first-line medications because of its significant side-effect profile, including increased risk of stroke, thromboembolism, and breast cancer [95].

One mechanism of this relationship is that estrogens upregulate bone morphogenic protein (BMP) signaling, which supports the differentiation of pre-osteoblasts into osteoblasts, aiding in the production of these bone-forming cells [86]. Osteocytes, derived from osteoblasts, are the most abundant cells in mature bone and play a key role in bone homeostasis and mechanosensing [12, 98]. Research has shown that estrogens exert an antiapoptotic effect on osteocytes through a gene transcription-dependent mechanism involving extracellular signal-regulated kinase (ERK) activation [99–101]. This antiapoptotic effect promotes the formation of bone, helping to prevent the balance of bone remodeling from excess resorption.

Another effect of estrogens is to attenuate the transcription of Receptor Activator of Nuclear factor Kappa-B Ligand (RANKL) and osteoprotegerin (OPG). RANKL is a key regulator of bone metabolism that binds the RANK receptor on osteoclast precursors, causing them to differentiate into mature osteoclasts. It is upregulated in mesenchymal lineage cells, T cells, and B cells under a lack of estrogens, promoting osteoclastogenesis and, if left unbalanced, to osteoporosis [86, 102, 103]. In contrast, estrogens stimulate production of OPG, which acts as a decoy receptor for RANKL to prevent its binding to the RANK receptor. Notably, levels of OPG are decreased under lack of estrogen, also promoting differentiation and activation of osteoclasts [86]. Estrogen deficiency also leads to an increase in pro-inflammatory molecules such as interleukin-1 (IL-1), IL-6, and tumor necrosis factor alpha (TNFα). These molecules are known to promote the activation of T cells, which in turn can induce osteoclast formation, contributing to bone loss in osteoporosis [86]. Higher peripheral concentrations of these cytokines have also been found in AD patients, further linking the two conditions through a common inflammatory response [104].

Estrogens have been implicated as a factor in both AD and osteoporosis through a multitude of different mechanisms including SHBG, signaling pathways, and inflammation. While better understood in the context of osteoporosis, the complexity of estrogen’s effects in AD is highlighted by the potential relationship between HRT timing and cognitive benefit. Conflicting evidence and partially understood mechanisms involving multiple hormones call for further research into this area.

## Neuroinflammation

Neuroinflammation, a complex and multifaceted response of the central nervous system (CNS) to injury, infection, or disease, plays a critical role in the pathogenesis of AD [31, 105]. This process is characterized by the activation of resident immune cells, primarily microglia and astrocytes, and the production of inflammatory mediators, which can contribute to neuronal damage and loss [105, 106].

The intricate role of neuroinflammation in AD has been underscored by numerous studies. Neuroinflammation has been observed to exacerbate the accumulation of Aβ plaques and promote the formation of neurofibrillary tangles, key pathological features of AD [105–107]. In a triple transgenic murine model of AD with mutant copies of APPswe, presenilin 1, and tauP301 L, general atrophy of hippocampal astroglia preceded Aβ plaque-related astrogliosis [106]. This suggests that neuroinflammation is not merely a consequence of AD but may actively contribute to its development and progression. Moreover, chronic neuroinflammation, characterized by sustained microglial activation and persistent exposure to proinflammatory cytokines, can lead to functional and structural changes in neurons, ultimately resulting in neuronal degeneration [31].

Recent research has highlighted the role of the triggering receptor expressed on myeloid cells 2 (TREM2) in AD. TREM2, a receptor expressed on microglial cells, is involved in the regulation of inflammatory responses within the CNS [108–110]. A novel variant in the gene encoding TREM2 has been identified (TREM2 R47H) that significantly increases risk of developing AD [108, 109]. This variant is believed to contribute to AD pathogenesis by enhancing oxidative stress and inflammation within the CNS [108]. Moreover, TREM2 deficiency in mice has been shown to attenuate tau pathology through a decreased neuroinflammatory response in multiple brain regions. This suggests that TREM2 signaling may play a role in the ability for microglia to respond to tau aggregates and contribute to their spreading [110]. However, conflicting evidence has shown that TREM2 knockout in 5xFAD mice leads to increased Aβ pathology [111] and that overexpression of TREM2 in APP/PS1 transgenic mice decreases neuroinflammation and Aβ accumulation [112]. Further studies are required to elucidate the true mechanism of the TREM2 receptor in AD pathology.

Interestingly, the TREM2 R47H variant has also been linked to gender-dependent changes in bone density [109]. A recent study found that female carriers of the TREM2 variant exhibited lower bone density compared to non-carriers, suggesting a potential link between AD and osteoporosis.

Neuroinflammation plays a pivotal role in the pathogenesis of AD, with the TREM2 variant serving as a key player in this process. The link between the TREM2 variant, AD, and osteoporosis provides a promising avenue for future research, potentially paving the way for novel therapeutic strategies targeting neuroinflammation in AD.

## Oxidative Stress

Many diseases, including AD and osteoporosis, have been linked to oxidative stress, which occurs when ROS overwhelm the antioxidant defenses of the body [57, 58, 103, 113–115]. Some common antioxidants that are produced by the body and ingested as nutrients through food are glutathione, vitamins C and A, polyphenols, and enzymes such as catalase [116].

In the context of AD, there are multiple mechanisms by which oxidative stress contributes to the pathophysiology of the disease. Oxidative stress causes DNA damage and protein misfolding, triggers neuronal apoptosis, compromises the function of neuronal mitochondria, and upregulates the production of Aβ and hyperphosphorylated tau, pathological hallmarks of AD [57, 58, 117, 118]. Additionally, oxidation of glycated proteins causes the accumulation of extracellular advanced glycation end products (AGEs). These are potent neurotoxins that bind to RAGE on the cell surface, producing proinflammatory molecules in a vicious positive feedback loop. This represents another mechanism by which RAGE contributes to the neuronal degeneration seen in AD [58].

There are also multiple ways in which oxidative stress has been linked to the activity of Aβ. Oxidative damage has been implicated in the impairment of glucose and glutamate transport and mitochondrial dysfunction induced by Aβ in synaptosomes [119]. In hippocampal cells, Aβ has been shown to increase the concentration of 4-hydroxynonenal (HNE), a product of lipid peroxidation [120]. HNE is neurotoxic, further supporting the role of oxidative stress in AD pathology. Aβ exposure can trigger neuronal apoptosis through activation of the JNK p38MAPK pathway. Simultaneous HNE and hydrogen peroxide (H_2_O_2_) treatment can fully mimic this trigger in vitro [121].

Oxidative stress also plays a significant role in bone remodeling and the development of osteoporosis. It has been shown to decrease differentiation of osteoblasts, as addition of H_2_O_2_ results in lower numbers of differentiation markers such as type 1 collagen and alkaline phosphatase, as well as decreased colony-forming unit-osteoprogenitor (CFU-O) formation [103, 114]. Osteocytes help to regulate bone remodeling through expression of sclerostin, a protein that negatively impacts bone formation by inhibiting osteoblast differentiation [103, 122–124]. Sclerostin and starvation-induced apoptosis are downregulated in osteocytes treated with antioxidants [124]. Additionally, ROS stimulates RANKL through ERK and NF-κB activation, leading to osteoclastogenesis [103]. These factors implicate oxidative stress in the dysregulation of bone homeostasis, favoring bone resorption over bone formation. To add to the effects of oxidative stress, menopause-related estrogen withdrawal may make bone more vulnerable to oxidative injury, increasing the risk of postmenopausal osteoporosis [113].

While the mechanisms involved in the effect of oxidative stress on these two diseases are different, the fact that they share this relationship provides an interesting avenue for future research and therapies.

## Therapies

The therapeutic landscape for AD is multifaceted, encompassing both non-pharmacological and pharmacological strategies. Initial interventions often involve lifestyle modifications, such as regular physical activity, a balanced diet, mental stimulation, and social engagement, which are recommended to delay cognitive decline. There are also many modifiable risk factors for AD such as hypertension, diabetes mellitus, and smoking. However, these modifications are often not sufficient to prevent or slow the progression of this disease [24, 125].

Transitioning to pharmacological treatments, cholinesterase inhibitors and N-methyl-D-aspartate (NMDA) antagonists have become mainstays in AD management. Cholinesterase inhibitors, including donepezil, rivastigmine, and galantamine, function by enhancing the levels of acetylcholine, a neurotransmitter integral to memory and learning processes, in the brain [126–128]. Glutamate, an excitatory neurotransmitter, is critical to synaptic plasticity and neuron survival through its interaction with the NMDA receptor. However, excessive NMDA activity can cause excitotoxicity and neuronal death. NMDA antagonists such as memantine block this receptor to suppress overactivity and help prevent neurodegeneration [129, 130].

In the realm of disease-modifying drugs, recent advancements have allowed for the utilization of monoclonal antibodies in the fight against AD. Aducanumab, a monoclonal antibody, targets soluble and insoluble Aβ peptides for degradation and has been shown to cause a significant reduction in Aβ plaques as well as clinically meaningful cognitive benefits [131]. Similarly, the monoclonal antibody lecanemab binds to soluble Aβ protofibrils, which are toxic to neurons. Recent evidence suggests that lecanemab reduces brain amyloid and slows disease progression. These drugs have both recently received approval from the FDA as treatment for AD [131, 132•].

Shifting focus to osteoporosis, therapeutic strategies aim to prevent bone loss, increase bone density, and reduce the risk of fractures. In contrast to AD, lifestyle modifications form the cornerstone of osteoporosis management. Regular weight-bearing exercise, adequate calcium and vitamin D intake, and smoking cessation are fundamental to maintaining bone health [133].

Treatments for osteoporosis include bisphosphonates, monoclonal antibodies, parathyroid hormone (PTH), and abaloparatide. Bisphosphonates, such as alendronate and risedronate, inhibit bone resorption, thereby maintaining BMD [134]. Denosumab, a monoclonal antibody, blocks RANKL to inhibit the development and activity of osteoclasts, thereby preserving BMD [134]. While these anti-catabolic medications are great for inhibiting the breakdown of bone, they do little to stimulate bone formation [134, 135].

PTH analogs such as teriparatide stimulate bone formation by activating osteoblasts [135, 136]. Abaloparatide, a synthetic analog of PTH-related protein, has shown potential in increasing BMD by enhancing bone formation while stimulating less expression of bone resorption factors such as RANKL than teriparatide [136]. A decrease in bone resorption is significant in this context, as PTH analogs have a limited anabolic window where the increase in bone formation exceeds the stimulation of resorption. Eventually, resorption starts to predominate, limiting the amount of time these medications can be used therapeutically [135, 136].

The common pathological features of both AD and osteoporosis include inflammation and oxidative stress, suggesting potential avenues for shared therapeutic strategies [31, 57, 58, 86, 109, 113]. Lifestyle modifications that promote overall health, such as regular physical activity and a balanced diet, are beneficial for both conditions [24, 125, 133].

The monoclonal antibody romosozumab binds and inactivates sclerostin, a glycoprotein secreted by osteocytes that inhibits osteoblast proliferation by blocking the Wnt signaling pathway [137]. This stimulates bone formation, and the drug has been shown to increase BMD and decrease vertebral fracture incidence in postmenopausal women [137, 138]. Lithium is another drug that has been shown to increase BMD and reduce fracture risk through activation of Wnt [139, 140]. The accepted mechanism is that lithium inhibits glycogen synthase kinase-3β (GSK-3β), a known inhibitor of the Wnt/β-catenin pathway [140]. GSK-3β is also believed to be involved in the hyperphosphorylation of tau proteins that causes AD pathology [141]. Studies have shown the ability of lithium to reduce AD pathology and slow cognitive decline in both mouse and human models [141–143]. These drugs highlight the shared Wnt signaling pathway as a potential dual therapeutic target.

Inhibition of FSH by an anti-FSH antibody has been shown to inhibit formation of Aβ plaques and NFTs in AD mouse models, leading to reversal of cognitive decline [97••]. A recent study used a humanized version of anti-FSH antibody named MS-Hu6 to increase bone formation in the femur and spine of mice [144•]. This novel therapy should be further explored in human subjects to evaluate its ability to target both diseases.

Moreover, the role of Aβ in both AD and osteoporosis opens possibilities for shared pharmacological interventions [14, 27, 29, 137]. Monoclonal antibodies like aducanumab and lecanemab, which target Aβ in the context of AD, could potentially be explored for their effects on bone health [131, 132•].

As we continue to unravel the complex interplay between AD and osteoporosis, further research is needed to develop therapeutic strategies that can effectively address both conditions. This dual approach not only promises to enhance our understanding of these diseases but also opens new avenues for comprehensive patient care.

## Conclusion

**Fig. 1:**
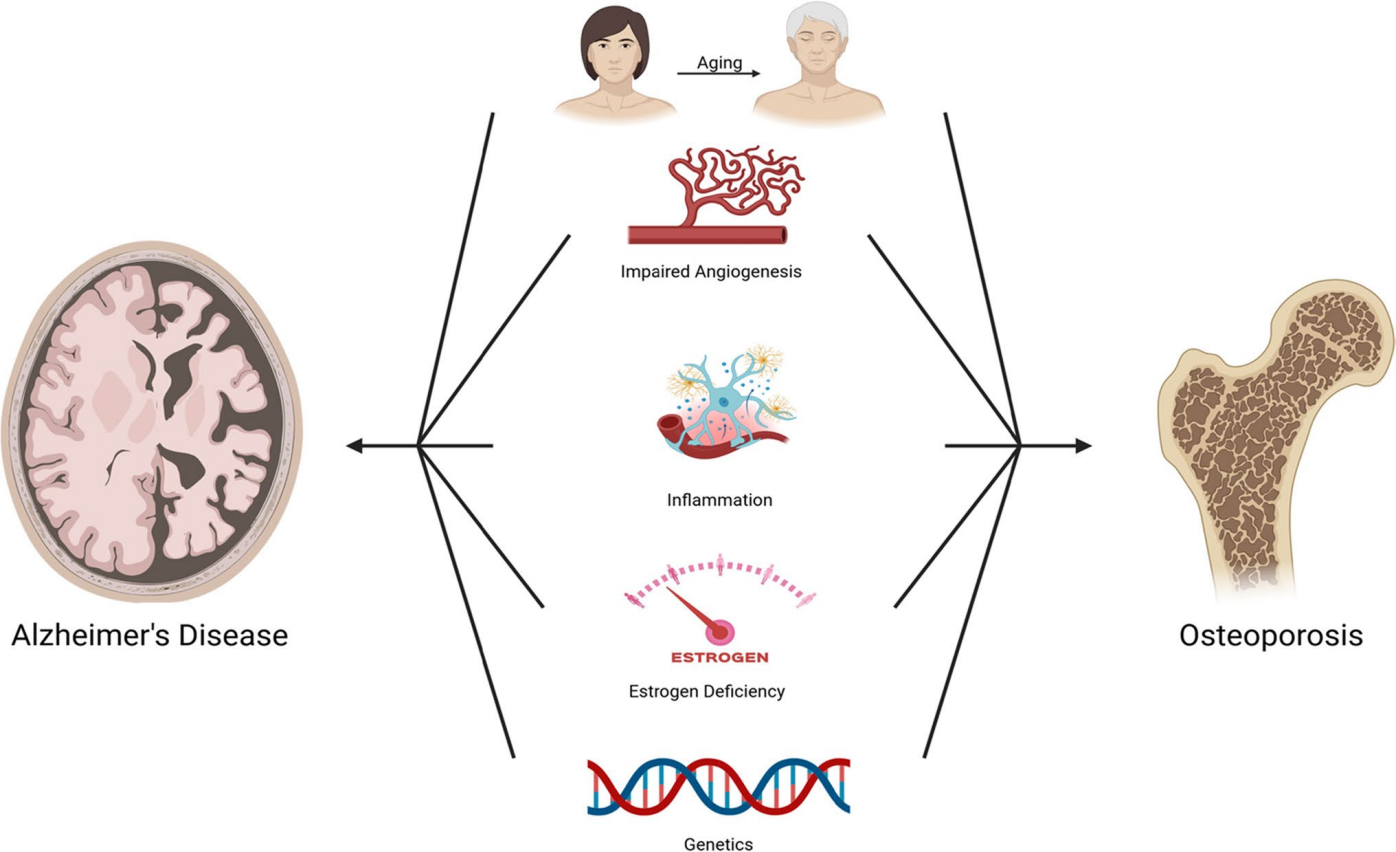
Factors such as aging, impairment of angiogenesis, inflammation, estrogen deficiency, and genetics can contribute to the pathogenesis of both Alzheimer’s disease and osteoporosis

In conclusion, the complex interplay between AD and osteoporosis reveals a bi-directional relationship, suggesting an underlying common pathology that affects both cognitive function and bone health. The interconnection between AD and osteoporosis is emphasized by the increased risk of fractures among individuals diagnosed with AD and, conversely, an increased risk of developing dementia or AD following fracture incidents. This relationship underscores the need for multifaceted approaches to treatment, including cognitive and gait function assessments and improvements in bone health management. As seen in Fig. 1, both diseases share risk factors, including age, genetics, inflammation, oxidative stress, and reduced estrogen levels, which impact the onset and progression of these conditions. In addition, AD and osteoporosis both utilize the Wnt/β-catenin signaling pathway, have impairments in angiogenesis, and are associated with the common genetic variant of TREM2, R47H, which can exacerbate neuroinflammation.

The range of therapeutic options for AD and osteoporosis is expanding, with both lifestyle and innovative pharmacological approaches. Lifestyle changes such as mental stimulation and regular physical activity can aid in slowing cognitive decline in AD but are typically not adequate for disease prevention. In contrast, smoking cessation, proper diet supplementation, and weight-bearing exercise are vital to osteoporosis treatment and prevention. Current pharmacological therapies for AD, like cholinesterase inhibitors, NMDA antagonists, and monoclonal antibodies targeting Aβ, work to mitigate the cognitive symptoms of AD. Osteoporosis treatments aim to preserve BMD and prevent fractures, utilizing therapies such as bisphosphonates, denosumab, and PTH analogs. Common pathological features of AD and osteoporosis open the possibility for shared therapeutic strategies, such as the potential use of monoclonal antibodies that target Aβ and activators of the Wnt/β-catenin signaling pathway to target both diseases. With HRT providing bone health benefits for postmenopausal women and potential cognitive benefits for perimenopausal or neurologically healthy women, it is intriguing to consider dual therapies for AD and osteoporosis, but proper studies would be required to evaluate the efficacy and timing of therapeutic benefits.

These findings urge a call for further research to fully understand the intricate interplay between these two conditions. Recognizing the complex link between AD and osteoporosis may not only aid in early diagnosis and treatment for each individual condition but also lead to the discovery of potential novel therapeutic strategies that could be beneficial for both conditions.
